# **α**-Mangostin Suppresses the Viability and Epithelial-Mesenchymal Transition of Pancreatic Cancer Cells by Downregulating the PI3K/Akt Pathway

**DOI:** 10.1155/2014/546353

**Published:** 2014-04-10

**Authors:** Qinhong Xu, Jiguang Ma, Jianjun Lei, Wanxing Duan, Liang Sheng, Xin Chen, Ang Hu, Zheng Wang, Zheng Wu, Erxi Wu, Qingyong Ma, Xuqi Li

**Affiliations:** ^1^Department of Hepatobiliary Surgery, First Affiliated Hospital of Medical College, Xi'an Jiaotong University, Xi'an, Shaanxi 710061, China; ^2^Department of Oncology, First Affiliated Hospital of Medical College, Xi'an Jiaotong University, Xi'an, Shaanxi 710061, China; ^3^Department of Pharmaceutical Sciences, North Dakota State University, Fargo, ND 58105, USA; ^4^Department of General Surgery, First Affiliated Hospital of Medical College, Xi'an Jiaotong University, Xi'an, Shaanxi 710061, China

## Abstract

**α**-Mangostin, a natural product isolated from the pericarp of the mangosteen fruit, has been shown to inhibit the growth of tumor cells in various types of cancers. However, the underlying molecular mechanisms are largely unclear. Here, we report that **α**-mangostin suppressed the viability and epithelial-mesenchymal transition (EMT) of pancreatic cancer cells through inhibition of the PI3K/Akt pathway. Treatment of pancreatic cancer BxPc-3 and Panc-1 cells with **α**-mangostin resulted in loss of cell viability, accompanied by enhanced cell apoptosis, cell cycle arrest at G1 phase, and decrease of cyclin-D1. Moreover, Transwell and Matrigel invasion assays showed that **α**-mangostin significantly reduced the migration and invasion of pancreatic cancer cells. Consistent with these results, **α**-mangostin decreased the expression of MMP-2, MMP-9, N-cadherin, and vimentin and increased the expression of E-cadherin. Furthermore, we found that **α**-mangostin suppressed the activity of the PI3K/Akt pathway in pancreatic cancer cells as demonstrated by the reduction of the Akt phosphorylation by **α**-mangostin. Finally, **α**-mangostin significantly inhibited the growth of BxPc-3 tumor mouse xenografts. Our results suggest that **α**-mangostin may be potentially used as a novel adjuvant therapy or complementary alternative medicine for the management of pancreatic cancers.

## 1. Introduction

Pancreatic ductal adenocarcinoma (PDAC) is one of the most malignant and lethal tumors, with an overall 5-year survival rate less than 5% [1]. The lethality of pancreatic cancer is largely due to the aggressive local invasion, metastases, and resistance to chemotherapy [2]. The poor prognosis in pancreatic cancer patients is closely correlated with the high proliferation and metastasis of tumour cells. Gemcitabine has been the standard first-line drug for patients with advanced pancreatic cancer since 1996, but it provides only a modest benefit to patients owing to the problems such as acquired chemoresistance and multiple adverse effects [3]. It is therefore clear that there is an urgent need to develop drugs that are more effective and have less toxicity compared to gemcitabine for the treatment of pancreatic cancer.

Mangosteen, a well-known tropical fruit, is native to Thailand and other tropical countries [4]. The purple pericarp of Mangosteen contains lots of healthy nutrients and pharmacologically active compounds, including xanthones, terpenes, anthocyanins, tannins, and phenols [5]. Since long time ago, the pericarp of this fruit has been used as a native drug by Southeast Asians to treat diseases, such as skin infections and wounds, amoebic dysentery, diarrhoea, and cholera [6]. In recent years, commercial products comprised of mangosteen, multiple vitamins and minerals, green tea, and other natural extracts are being widely recommended to cancer patients as a dietary supplement [7]. Although there is no sufficient clinical evidence that mangosteen could suppress the growth of tumors or reduce the incidence of malignancies, the commercial products are one of the best-selling botanical dietary supplements [8].

A class of compounds known as xanthones isolated from mangosteen possess a wide range of biological activities including anti-inflammatory [9, 10], neuroprotective [11], cardioprotective [12, 13], and antioxidant activity [7, 14, 15]. *α*-Mangostin is one of the major bioactive and most abundant xanthones derived from mangosteen [16]. *α*-Mangostin, as a chemopreventive and chemotherapeutic bioactive compound, has been widely investigated [17]. It was shown that *α*-mangostin has a potential inhibitory effect on several carcinomas. It could induce cycle arrest of cancer cell [18], inhibit cell viability [19], induce apoptosis and differentiation [20], reduce inflammation, and decrease adhesion [21–23], invasion [24], and metastasis of cancer cells [23, 25]. However, the underlying pharmacology of its antitumor effect is largely unknown.

In this study, we have investigated the effects of *α*-mangostin on the cell viability and epithelial-mesenchymal transition in pancreatic cancer BxPc-3 and Panc-1 cells. We have also explored the underlying potential molecular mechanisms and found that the PI3K/Akt pathway was inhibited by *α*-mangostin.

## 2. Materials and Methods

### 2.1. Reagents

*α*-Mangostin (purity > 98%) was obtained from Sigma (CA, USA) and dissolved in dimethylsulfoxide (DMSO; St. Louis, MO, USA) at the stock concentration of 100 mM. Recombinant human TGF-*β*1 was purchased from Zhongshan Golden Bridge Biotechnology (Beijing, China). Millicell culture plate inserts and Matrigel were purchased from Millipore (Bedford, MA, USA). Antibodies against Bcl-2 or *β*-actin were from Santa Cruz Biotechnology (Santa Cruz, CA, USA); anticleaved caspase-3, cyclin-D3, cyclin-D1, Akt, and phospho-Akt (Ser473) antibodies were purchased from Cell Signaling Technology; anti-MMP-2 and anti-MMP-9 antibodies were from Proteintech (USA); antibodies against E-cadherin, N-cadherin, and vimentin were procured from Bioworld (Minneapolis, MN, USA).

### 2.2. Cell Culture

BxPc-3 and Panc-1 cells were purchased from Chinese Academy of Sciences Cell Bank of Type Culture Collection (CBTCCCAS); hTERT-HPNE cell was purchased from ATCC (Manassas, VA) and cultured at 37°C and 5% CO_2_ in RPMI-1640 and Dulbecco's modified Eagle's medium (DMEM/High Glucose) (HyClone, Logan, USA) supplemented with 10% fetal bovine serum (FBS) (HyClone, Logan, USA), 100 *μ*g/mL ampicillin, and 100 *μ*g/mL streptomycin.

### 2.3. Cell Viability Assay

BxPc-3, Panc-1, and hTERT-HPNE cells at 50–60% confluency were cultured in medium supplemented with 1% FBS for 24 h to get synchronized G1 phase cells, plated into 96-well plates at a density of 1 × 10^4^ cells per well, and incubated overnight in medium supplemented with 10% FBS. Cells were then treated with various concentrations of *α*-mangostin in 0.1% DMSO or with 0.1% DMSO alone as control. Following incubation for 6, 12, 24, and 48 h at 37°C, relative cell viability was quantified by MTT assay as previously reported [26]. 20 *μ*L of 5 mg/mL MTT (Sigma, St. Louis, MO, USA) was added into each well after media were removed and incubated at 37°C for 4 h. Then, 150 *μ*L DMSO was added to each well and the optical density (OD) was measured at 492 nm on a Multifunction Microplate Reader (POLARstar OPTIMA; BMG, Offenburg, Germany). The proliferation inhibition rate was calculated according to the equation: proliferation inhibition rate = (1 − OD  sample/OD  control) × 100%.

### 2.4. Cell Cycle Analysis

Cells (1 × 10^5^ cells/mL) were seeded in six-well plates coated with 1% gelatin and allowed to grow to 80% confluency. Then, the media were replaced with fresh media containing various different concentrations of *α*-mangostin. After 24 h of incubation, the cells were fixed in 70% alcohol for 30 min on ice. Cells were treated with RNase A (Sigma) at 37°C and stained with propidium iodide in the dark for 30 min. DNA content was assayed on a FACSCalibur (BD, Franklin Lakes, NJ) and cell cycle analysis was conducted using CellQuest software.

### 2.5. Apoptosis Assay

Apoptosis was assessed with an Annexin V-FITC/PI apoptosis detection kit (Beyotime Institute of Biotechnology, Shanghai, China) according manufacturer's instructions. Cells were seeded (10^5^/well) in 6-well plates in DMEM supplemented with 1% FBS. Twenty-four hours later, the medium was replaced with fresh medium containing various concentrations of *α*-mangostin. After additional 24 h, cells were trypsinized, washed with PBS, and stained with Annexin V and propidium iodide in the dark. The percentage of apoptotic cells was quantified by flow cytometry.

### 2.6. Cell Migration and Invasion Assays

Cell invasion and migration assay were performed using Transwell chambers (Millipore) as previously described [27]. The 8 *μ*m pore inserts were coated with or without Matrigel (Sigma-Aldrich) for invasion or migration assays. Cell suspensions containing 1% FBS (200 *μ*L, 5 × 10^4^ cells) were seeded into the upper chamber and 500 *μ*L DMEM containing 20% FBS was added in the lower chamber. Noninvading cells were removed with a cotton-tipped swab after 24 h incubation, and the invading cells on the bottom surface of membrane were stained with 0.1% crystal violet. The invading cell numbers were quantified by counting 10 random fields at ×200 magnification.

### 2.7. Western Blotting

Total proteins were extracted by RIPA Lysis Buffer (Beyotime, Guangzhou, China) according to the manufacturer's instruction. Western blotting was performed as previously described [28]. In brief, proteins were separated on a 10% SDS-PAGE gel and transferred onto PVDF membranes (Roche). Membranes were blocked with 5% nonfat dry milk in Tris-Buffered Saline and Tween 20 (TBST) at room temperature for 1 h and incubated overnight at 4°C with the following primary antibodies: anti-Bcl-2, anticleaved caspase-3, anti-cyclin-D3, anti-cyclin-D1, anti-MMP-2 and anti-MMP-9. After incubation with HRP-conjugated secondary antibodies for 2 h at room temperature, immunoreactive bands were developed by enhanced chemiluminescence. *β*-Actin was stained as loading control.

### 2.8. Real-Time PCR

Total RNA was extracted by Fastgen200 RNA isolation system (Fastgen, Shanghai, China) as the manufacturer's protocol. Total RNA was reverse-transcribed into cDNA using the PrimeScript RT reagent kit (Takara, Dalian, China). qRT-PCR was conducted using the iQ5 Multi-color Real-Time PCR Detection System (Bio-Rad, Hercules, CA) and SYBR Premix Ex Taq II (Takara, Dalian, China) as previously described to quantify mRNA levels of MMP-2, MMP-9, E-cadherin, N-cadherin, and vimentin [29]. The comparative C (T) method was used to quantitate the expression of each target gene using *β*-actin as the normalization control. The PCR primer sequences for MMP-2, MMP-9, E-cadherin, N-cadherin, vimentin, and *β*-actin are listed in Supplemental Information Table S1 (see Supplemental Information Table S1 available online in Supplementary Materials at http://dx.doi.org/10.1155/2014/546353).

### 2.9. Subcutaneous Xenografts* In Vivo*

Male BALb/c nude mice were purchased and housed in the Animal Center at Medical College, Xi'an Jiaotong University. Twenty animals were randomly divided into four groups, with five animals in each group. The animals in group 1 received vehicle (100 *μ*L saline) by oral gavage and served as control. The animals in groups 2 and 3 received *α*-mangostin suspension (dissolved in 100 *μ*L saline and 50 or 100 mg/kg) by oral gavage five times weekly [18]. Body weights were recorded once weekly throughout the study. According to the previous report [27], 2 × 10^6^ cells (100 *μ*L) in a 50% Matrigel mixture were injected subcutaneously in nude mice at four to six weeks old. The dimensions, length (*l*), and width (*w*) of the tumors were measured with Vernier calipers every 3 days. Tumor volume was calculated using the equation (*l* × *w* ^2^)/2. The mice were euthanized 30 days after the subcutaneous injection.

### 2.10. Ethics Statement

All* in vivo* experimental protocols were evaluated and approved by the Animal Care and Use Committee of College of Medicine, Xi'an Jiaotong University.

### 2.11. Statistical Analysis

Each experiment was performed at least for three times. Data are presented as means ± standard deviation. Differences were evaluated using a* Student's t*-test, with *P* < 0.05 considered to be statistically significant.

## 3. Results

### 3.1. Treatment with *α*-Mangostin Results in Loss of Viability of Pancreatic Cancer Cells

**Figure 1:**
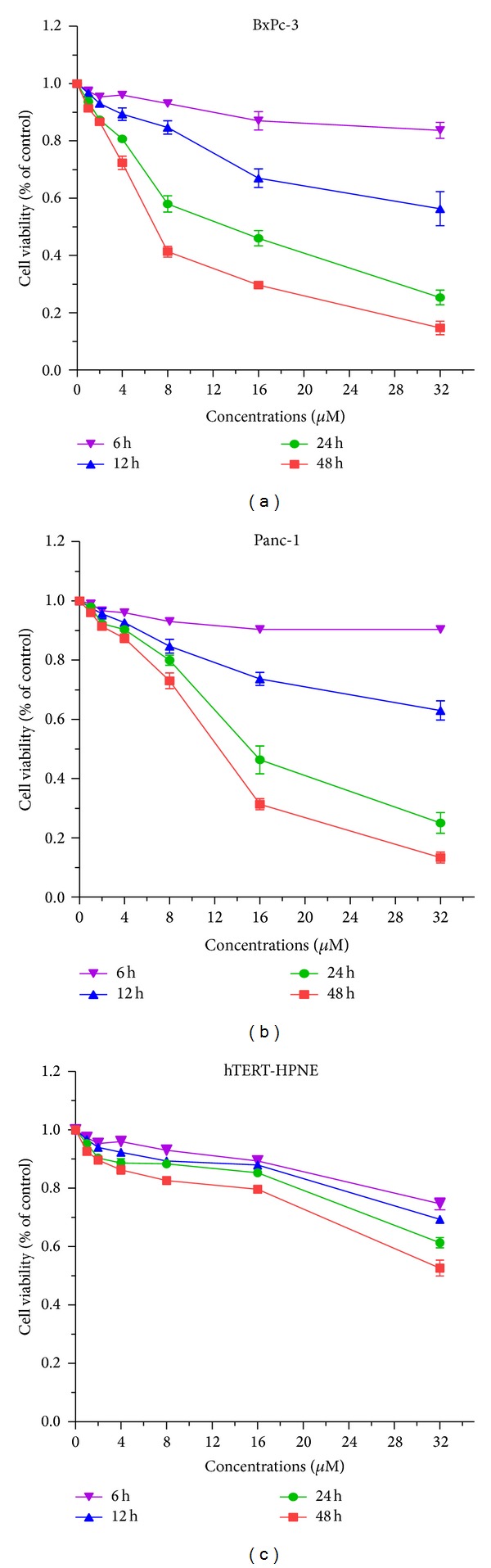
Treatment of pancreatic cancer cells with *α*-mangostin results in loss of cell viability. Pancreatic cancer BxPc-3 (a), Panc-1 (b), and hTERT-HPNE (c) cells were treated with *α*-mangostin at the indicated concentrations for 6, 12, 24, and 48 h. Cell viability relative to control was assessed using the MTT assay.

To investigate the effect of *α*-mangostin on the viability of pancreatic cancer cells, we treated BxPc-3, Panc-1, and hTERT-HPNE cells (normal human pancreatic ductal epithelial cell) with different concentrations of *α*-mangostin (0, 2, 4, 6, 8, 16, and 32 *μ*M) for 6, 12, 24, and 48 h and assessed cell viability by the MTT assay. Treatment with *α*-mangostin at concentrations of more than 8 *μ*M significantly reduced the viability of both cancer cell lines in a time-dependent manner (*P* < 0.05) (Figures 1(a) and 1(b)); however, limited inhibitory effect on hTERT-HPNE cells was observed (Figure 1(c)). Treatment with 32 *μ*M *α*-mangostin resulted in more than 80% loss of cell viability in both pancreatic cancer cell lines and 50% in hTERT-HPNE cells after 48 h.

### 3.2. *α*-Mangostin Induces Apoptosis of Pancreatic Cancer Cells

**Figure 2:**
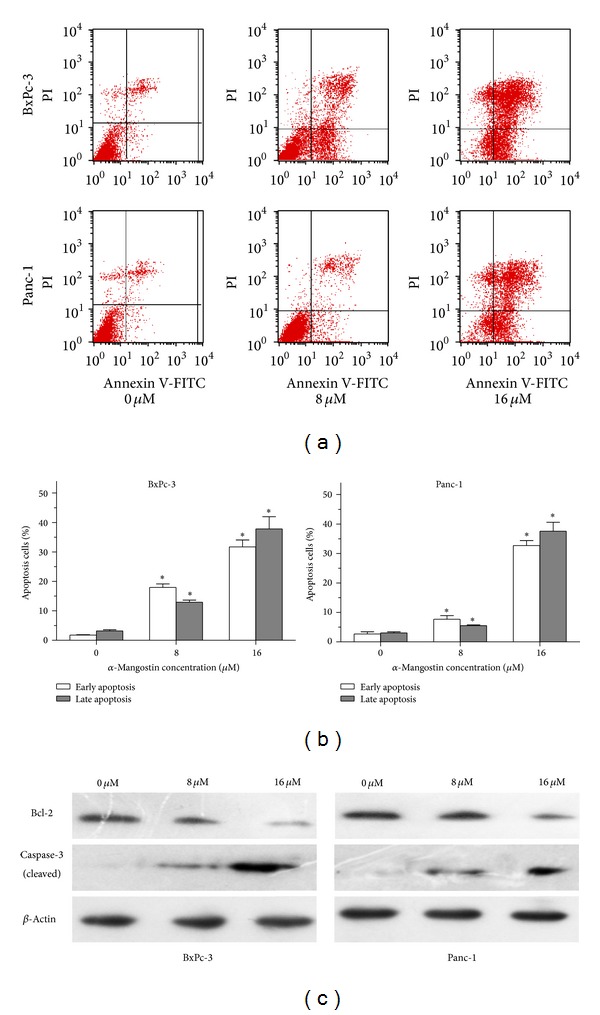
*α*-Mangostin induces apoptosis of pancreatic cancer cells. (a) Panc-1 and BxPC-3 cells were treated with *α*-mangostin (0 *μ*M, 8 *μ*M, or 16 *μ*M) for 24 h. Apoptotic cells were evaluated by Annexin V-FITC/PI staining and flow cytometry. Representative FACS plots are shown. (b) Apoptosis rate of the early and late apoptosis was quantified by flow cytometry. *P < 0.05 compared with controls. (c) Protein levels of Bcl-2 and cleaved caspase-3 were detected by Western blotting with *β*-actin as loading control.

To address the underlying mechanism of the inhibitory effect of *α*-mangostin on pancreatic cancer cell viability, we measured *α*-mangostin-induced apoptosis in BxPc-3 and Panc-1 cells by flow cytometry. As shown in Figures 2(a) and 2(b), treatment with 8 *μ*M *α*-mangostin resulted in increased early (Annexin V-FITC) and late (PI) apoptotic cells compared to untreated controls. Notably, when compared to the untreated group, more than 30% cells of both of the cell lines underwent apoptosis following treatment of 16 *μ*M *α*-mangostin (Figures 2(a) and 2(b), *P* < 0.05). Consistent with these results, immunoblots demonstrated that *α*-mangostin caused a dose-dependent reduction in the antiapoptosis Bcl-2 protein levels and an increase in the levels of cleaved of caspase-3, a marker of cell apoptosis (Figure 2(c)). These results clearly demonstrate that *α*-mangostin induces apoptosis of pancreatic cancer cells.

### 3.3. *α*-Mangostin Induces Cell Cycle Arrest at the G1/G0 Phase in Pancreatic Cancer Cells

**Figure 3:**
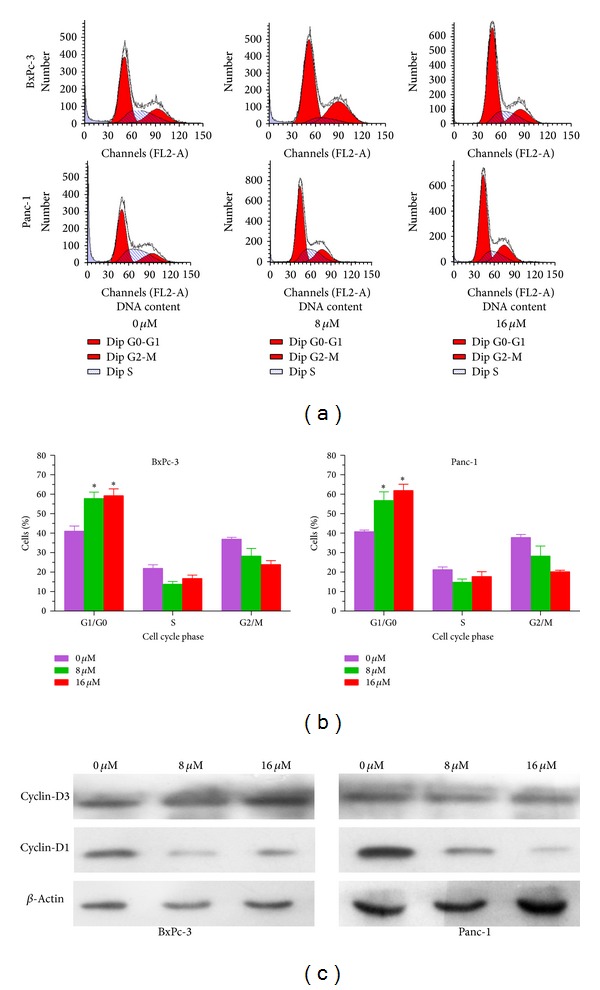
*α*-Mangostin induces cell cycle arrest in pancreatic cancer cells. (a) The effect of *α*-mangostin on cell cycle progression in pancreatic cancer cells was assessed by flow cytometry. Cells were fixed, stained, and analyzed for DNA content. (b) The distribution and percentage of cells in G1/G0, S and G2/M phase of the cell cycle are indicated. (c) Protein levels of cyclin-D3 and cyclin-D1 were assessed by Western blotting with *β*-actin as loading control.

Most drugs inhibit cell growth and promote cell apoptosis through inducing cell cycle arrest. To test whether *α*-mangostin induces cell cycle arrest of pancreatic cancer cells, we treated BxPc-3 and Panc-1 cells with different concentrations of *α*-mangostin for 24 h and performed cell cycle analysis by PI/flow cytometry. As shown in Figures 3(a) and 3(b), pancreatic cancer cells treated with 8 *μ*M *α*-mangostin significantly accumulated in the G1/G0 phase, while there were more cells in G1/G0 phase after treatment with 16 *μ*M *α*-mangostin. In consistence, *α*-mangostin treatment led to a reduction of cells in S and G2/M phases (Figures 3(a) and 3(b)). The increase of G1/G0 phase in conjunction with the decrease of S and G2/M phases suggests that the reduction of cell viability may be due at least in part to the induction of cell cycle arrest by *α*-mangostin.

Cyclin D-dependent kinases 4 and 6 (CDK4/6) are the main drivers for the transition of cells from G1 to S phase. Indeed, in BxPc-3 cells, 8 *μ*M *α*-mangostin slightly and 16 *μ*M *α*-mangostin markedly reduced the protein level of cyclin-D1 after 24 h of treatment (Figure 3(c)). In Panc-1 cells, 8 *μ*M and 16 *μ*M *α*-mangostin dramatically decreased the protein level of cyclin-D1 (Figure 3(c)). In both cell lines, the effects of *α*-mangostin on the protein levels of cyclin-D3 were marginal. These results indicate that *α*-mangostin inhibits cell cycle progression by downregulating cyclin-D1.

### 3.4. *α*-Mangostin Inhibits the Migration and Invasion of Pancreatic Cancer Cells

**Figure 4:**
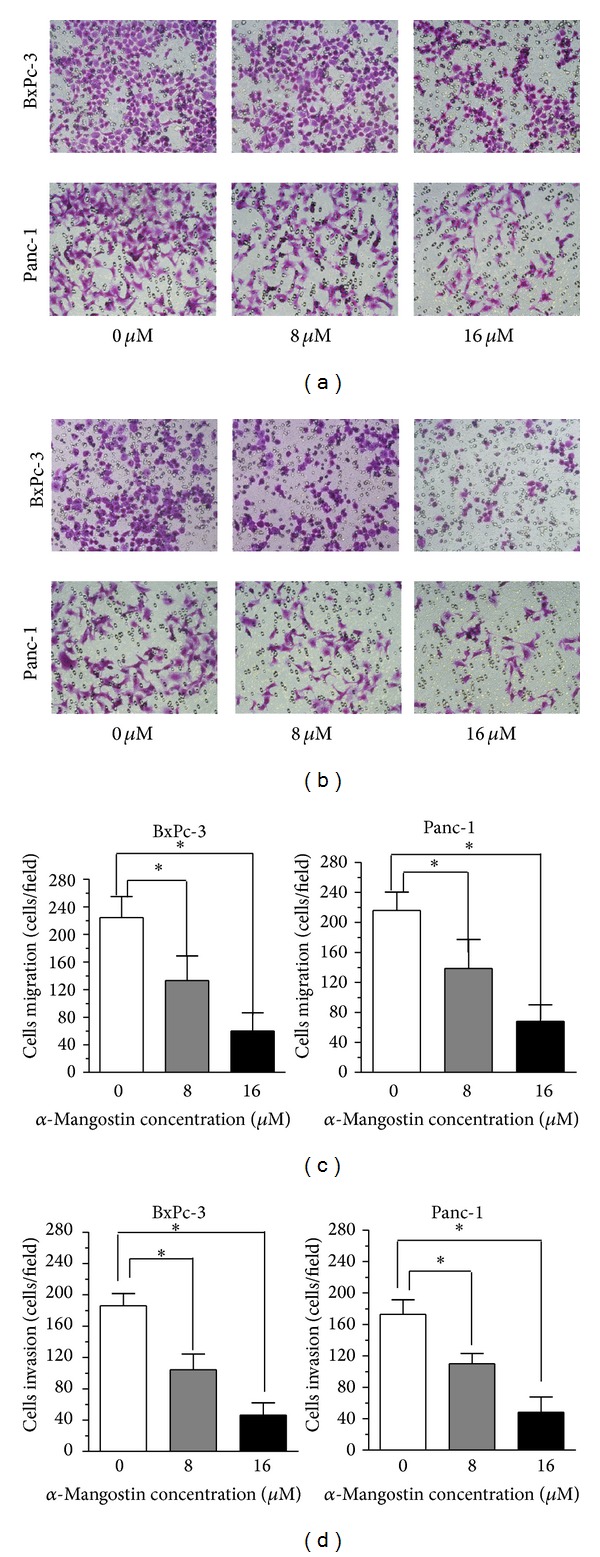
*α*-Mangostin inhibits the migration and invasion of pancreatic cancer cells. (a) The effects of *α*-mangostin on pancreatic cancer cell migration capability were assessed by Transwell assay. (b) The number of migrated cells was quantified by counting the number of cells from 10 random fields at ×200 magnification. (c) The effect of *α*-mangostin on pancreatic cancer cell invasion capability was assessed by Matrigel invasion assay. (d) The number of migrated cells was quantified by counting the number of cells from 10 random fields at ×200 magnification. *P < 0.05 compared with controls.

**Figure 5:**
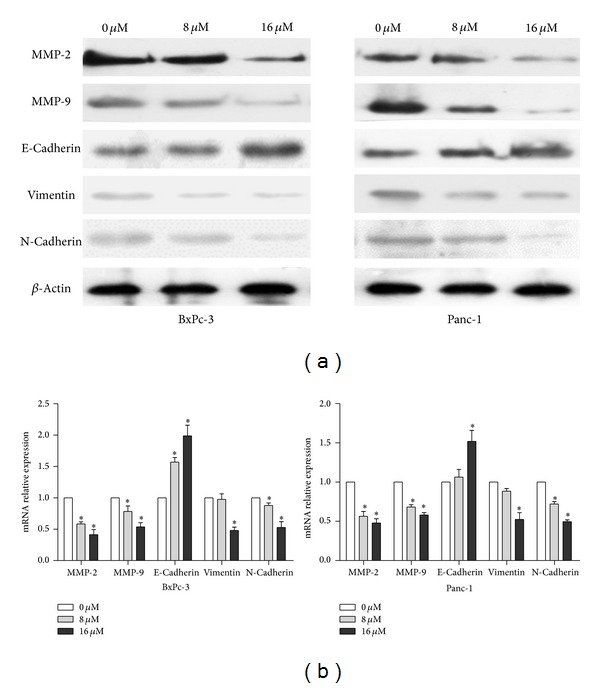
*α*-Mangostin modulates the expression of EMT-related genes in pancreatic cancer cells cells. (a) Panc-1 and BxPC-3 cells were treated with *α*-mangostin 16 *μ*M at the indicated concentrations for 24 h. Protein (a) and mRNA (b) levels of MMP-2, MMP-9, E-cadherin, vimentin, and N-cadherin were measured by Western blotting and qRT-PCR, respectively. *P < 0.05 compared with controls.

To evaluate the effect of *α*-mangostin on pancreatic cancer cell migration, we performed Transwell assays and found that *α*-mangostin at concentrations of 8 *μ*M and 16 *μ*M significantly inhibited the migration of BxPc-3 and Panc-1 cells (Figure 4(a)). Next, we assessed the effect of *α*-mangostin on pancreatic cancer cell invasion using a Matrigel invasion assay. *α*-Mangostin significantly suppressed the ability of these cells to invade through the Matrigel in a dose-dependent manner (Figure 4(b)). Moreover, *α*-mangostin reduced both the mRNA (Figure 5(a)) and protein levels (Figure 5(b)) of MMP-2 and MMP-9, which play critical roles in migration, invasion, and metastasis of cancer cells. These observations demonstrate that *α*-mangostin inhibits the migration and invasion of pancreatic cancer cells* in vitro*.

### 3.5. *α*-Mangostin Modulates the Expression of EMT-Related Genes in Pancreatic Cancer Cells

The inhibition of cell migration and invasion of pancreatic cancer cells suggests that *α*-mangostin may affect EMT of cancer cells. To test this, we measured the expression of EMT related-genes by immunoblotting and qRT-PCR. *α*-Mangostin treatment of both BxPc-3 and Panc-1 cells resulted in a concentration-dependent increase in the protein levels of the epithelial marker E-cadherin and a decrease in the protein levels of the mesenchymal markers vimentin and N-cadherin (Figure 5(a)). Similar alteration of the mRNA levels of these corresponding genes by *α*-mangostin was also observed (Figure 5(b)). These results further support the observation that *α*-mangostin has an inhibitory effect on the acquisition of mesenchymal-like characteristics by pancreatic cancer cells and consequently suppresses their capacity for dissemination and invasion.

### 3.6. *α*-Mangostin Suppresses the Activation of the PI3K/Akt Pathway in Pancreatic Cancer Cells

**Figure 6:**
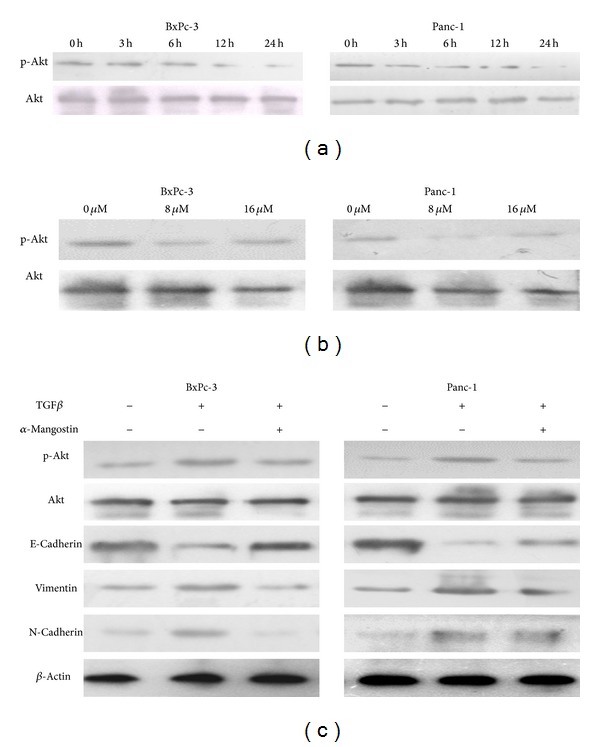
*α*-Mangostin suppresses activation of the PI3K/Akt pathway in pancreatic cancer cells. Protein levels of total Akt and pAkt-S473 were detected by Western blotting. (a) Panc-1 and BxPC-3 cells were treated with *α*-mangostin (16 *μ*M) for 0 h, 3 h, 6 h, 12 h, and 24 h. (b) Panc-1 and BxPC-3 cells were treated with *α*-mangostin at the indicated concentrations for 24 h. (c) Panc-1 and BxPC-3 cells were treated with 5 ng/mL TGF-*β* either alone or in combination with 16 *μ*M *α*-mangostin for 24 h. Protein levels of total Akt, pAkt-S473, E-cadherin, vimentin, and N-cadherin were measured by Western blotting with *β*-actin as loading control.

The PI3K/Akt pathway, one of the predominant cell growth-promoting signaling pathways, enhances cell proliferation mainly by increasing D-type cyclins. The arrest of cell cycle in the G1 phase and downregulation of cyclin-D1 (Figure 3) suggest that *α*-mangostin may downregulate the activity of the PI3K/Akt pathway in pancreatic cancer cells. To test this hypothesis, we assessed the phosphorylation of Akt at ser473 by immunoblotting of BxPc-3 and Panc-1 cells treated with *α*-mangostin. Our results revealed that *α*-mangostin reduced the phosphorylation of Akt at ser473 (Figure 6(a)). We also observed that Akt phosphorylation was significantly decreased after treatment with the *α*-mangostin in a dose- and time-dependent manner (Figures 6(a) and 6(b)).

The deregulation of PI3K/Akt pathway is associated with enhanced cell migration, invasion, and metastasis of cancer cells. Moreover, a recent study demonstrated that TGF-*β* induces epithelial to mesenchymal transition through the activation of PI3K/Akt/mTOR pathway [30]. These facts led us to hypothesize that *α*-mangostin may suppress the migration, invasion, and EMT in pancreatic cancer cells through dampening the PI3K/Akt pathway. We therefore compared the TGF-*β*-induced phosphorylation of Akt and upregulation of EMT-related genes [31] in BxPc-3 and Panc-1 cells treated with 5 ng/mL TGF-*β* [31] with or without 16 *μ*M of *α*-mangostin. While TGF-*β* enhanced the phosphorylation of Akt and the expression of vimentin and N-cadherin, these TGF-*β*-induced effects were significantly abolished by *α*-mangostin (Figure 6(b)). These results demonstrate that *α*-mangostin induces multiple biological effects in pancreatic cancer cells through inhibition of the PI3K/Akt pathway.

### 3.7. *α*-Mangostin Suppresses the Growth of Human Pancreatic Cancer BxPc-3 Cells* In Vivo*

**Figure 7:**
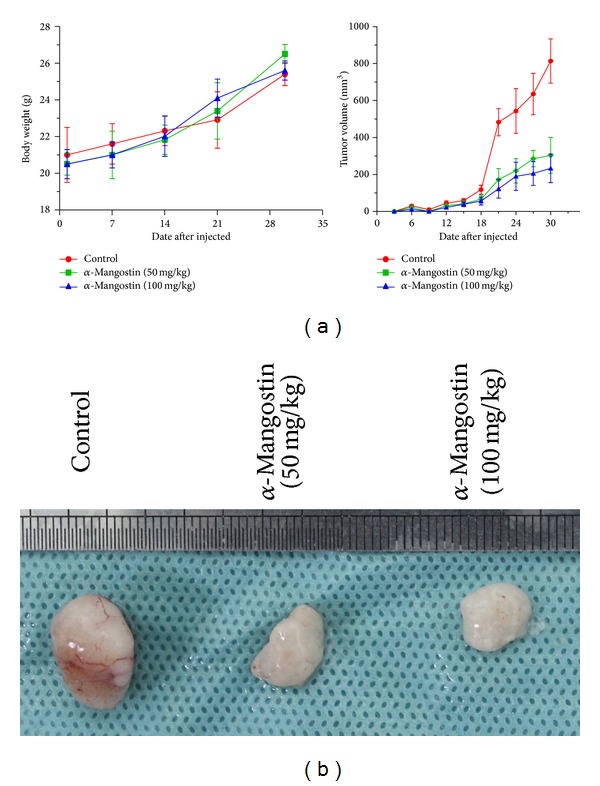
*α*-Mangostin suppresses the* in vivo* growth of human pancreatic cancer cells. (a) Body weight was measured weekly and tumor volumes (mm^3^) were calculated every 3 days throughout the study. (b) Effects of *α*-mangostin on the growth of xenograft tumors. Photographs of subcutaneous xenograft tumors derived from BxPc-3 cells in nude mice with (left) or without (right) *α*-mangostin treatment (50 and 100 mg/kg) on day 30 after tumor cell injection.

To extend our* in vitro* observations to the* in vivo* context, we conducted* in vivo *tumor xenograftexperiments. BALb/c nude mice were implanted with BxPc-3 cells and divided into three cohorts, one of which received vehicle and the others *α*-mangostin (50 or 100 mg/kg). We found a dramatic difference in the growth of tumor xenografts with 50 mg/kg *α*-mangostin, and more significant inhibitory effect of 100 mg/kg was observed (*P* < 0.05; Figure 7(a)). The average tumor volume of the group treated with *α*-mangostin was significantly lower on day 30 after tumor cell injection compared with that of the control group (Figure 7(b)). No differences in body weights were found between the mice with or without *α*-mangostin during the study. These data showed that *α*-mangostin suppresses the growth of tumor xenografts derived from pancreatic cancer cells and has no harmful effects on the animals.

## 4. Discussion

In this study, we observed that *α*-mangostin inhibited cell viability and induced cell apoptosis, which were accompanied by the activation of caspase-3 and decreased levels of Bcl-2 in BxPc-3 and Panc-1 cells. Moreover, *α*-mangostin promoted cell cycle arrest and inhibited cell invasion, which were associated with the decreased expression of cyclin-D and MMPs. Furthermore, we found that *α*-mangostin suppressed the EMT progression and activity of the PI3K/Akt pathway in pancreatic cancer cells. The subcutaneous tumor xenograft model showed that the oral administration of *α*-mangostin resulted in significant suppression of the growth of tumor xenografts in nude mice. Our findings suggest that *α*-mangostin is a potential drug for adjuvant therapy or a complementary alternative medicine for the management of pancreatic cancers.

Despite great advances in modern medicine during the past two decades, pancreatic cancer is still associated with an extremely high mortality rate (approaching 100%) [2]. Currently, Gemcitabine is considered as a first-line drug for pancreatic cancer, but its efficacy is still disappointing [32] and the pancreatic cancer survival has not improved substantially over the past 25 years. Recently, the dietary and synthetic agents purported to possess greater efficacy and lower toxicity have attracted great attention for the prevention and treatment of pancreatic cancer [33].

Previous studies showed that *α*-mangostin could inhibit the growth and induce the apoptosis of human prostate cancer [18] and colon cancer [34] cells. Similarly, we found that *α*-mangostin inhibited the proliferation and induced apoptosis of pancreatic cancer cells in a time- and dose-dependent manner, but limited inhibitory effect on the normal human pancreatic ductal epithelial cell was observed. The caspase-3 cascade is activated by proapoptotic molecules such as cytochrome c when released from mitochondria and is inhibited by antiapoptotic members of the Bcl-2 proteins. We demonstrated that *α*-mangostin promoted the activation of caspase-3 but decreased Bcl-2, suggesting that *α*-mangostin may potentially act on mitochondria and induce apoptosis.

*α*-Mangostin has been previously reported to induce cell cycle arrest in breast cancer cells [35] and colon cancer cells [34]. Consistent with these studies, our results demonstrated that *α*-mangostin significantly inhibited the ability of cells to transit from G1 to the S phase. The transition from the G1 phase to S phase is an important cell cycle control point. Cyclin-D1 is a critical cell cycle regulatory molecule and is required for the cell cycle progression through G1 to S phase [36]. The growth stimulatory signals received by the cells in the early stage of G1 phase increase the levels of cyclin-D1. It was reported that *α*-mangostin for 24 h suppressed cyclin-D1 which was followed by apoptosis in prostate cancer cells [18]. In agreement with this report, we observed that *α*-mangostin promoted cell cycle arrest at G1 phase and downregulated cyclin-D1 in BxPc-3 and Panc-1 cells. These results indicate that the inhibitory effect of *α*-mangostin on the pancreatic cancer cell might be due to the downregulation of cyclin-D1 expression and a consequent delay in the G1/S transition.

Migratory and invasive abilities are important characteristics of metastatic cancer cells. Epithelial-mesenchymal transition is the process wherein epithelial cells acquire fibroblast-like properties and exhibit reduced cell-cell adhesion and increased motility. During oncogenesis, EMT may endow cancer cells with enhanced motility and invasiveness. Recent studies showed that *α*-mangostin could inhibit the metastasis and invasion in skin, prostrate, breast, and lung [25]. In the present study, *α*-mangostin was demonstrated to decrease the migration and invasion of pancreatic cancer cells. In addition, we found that *α*-mangostin modulated the expressions of invasion-related (MMP-2 and MMP-9) and EMT-associated (E-cadherin, vimentin and N-cadherin) genes. Our results suggest that *α*-mangostin may inhibit migration and invasion of pancreatic cancer cells by downregulating MMPs and inhibiting EMT progression.

Dysregulation of the PI3K/Akt pathway is quite common in PDAC. Up to 60% of PDAC tissues and most PDAC cell lines exhibit increased AKT activity [37]. The serine/threonine kinase Akt, the most studied signaling molecule downstream of PI3K, is involved in the stimulation of cell proliferation, inhibition of apoptosis, alteration of the cell cycle, and promotion of invasiveness as well as induction of EMT [38–40]. These findings prompted us to hypothesize that the biological action of *α*-mangostin may be mediated via PI3K/Akt pathway. Our results showed that *α*-mangostin reduced the basal activity of Akt and abolished the TGF-*β*-induced Akt phosphorylation.

The anticancer potential of *α*-mangostin was further supported by our* in vivo* studies that involve generation of subcutaneous xenograft tumors derived from pancreatic cancer cells in nude mice. A previous study showed that pretreatment of rats with *α*-mangostin (200 mg/kg body wt.) orally for up to 8 days had no observable adverse effects in solid organ systems [13]. Another report demonstrated that F344 rats fed with custom-blended food pellets that contained 0.02 or 0.05% *α*-mangostin for 5 weeks also showed no apparent adverse effects [41]. In addition, mangosteen that contains abundant amounts of *α*-mangostin is widely consumed by humans in the form of juices, dietary supplements, and fruit [18]. Therefore, the anticancer activity of *α*-mangostin suggests that *α*-mangostin has the potential to be a novel adjuvant therapy or complementary alternative medicine for the management of pancreatic cancer.

## 5. Conclusions

The present study demonstrated that *α*-mangostin suppressed the proliferation, migration, and invasion as well as EMT of pancreatic cancer cells. These multiple biological effects might result from the suppression of the PI3K/Akt signaling pathway by *α*-mangostin. These results suggest that *α*-mangostin is a potential anticancer agent for the treatment of pancreatic cancer.

## Supplementary Material

Supplementary Table S1: PCR Primer Sequences for Human *β*-Actin, CXCL12, CXCR4, NGF, uPA, MMP-2 and Rat CXCL12, CXCR4.
