# Supplementary material for: α-Mangostin Suppresses the Viability and Epithelial-Mesenchymal Transition of Pancreatic Cancer Cells by Downregulating the PI3K/Akt Pathway
Source: Biomed Res Int. 2014 Apr 10;2014:546353. doi: 10.1155/2014/546353 (PMC4000937; doi:10.1155/2014/546353)
Supplement: Supplementary file 1 — Supplementary Table S1: PCR Primer Sequences for Human β-Actin, CXCL12, CXCR4, NGF, uPA, MMP-2 and Rat CXCL12, CXCR4. [file 546353.f1.pdf]

Table S1. PCR Primer Sequences

genes	Primer Sequence
Human	
β-actin	P1: 5’- AGCGAGCATCCCCCAAAGTT -3’
	P2: 5’- GGGCACGAAGGCTCATCATT -3’
MMP-2	P1: 5’- GATGATGCCTTTGCTCGTGC -3’
	P2: 5’- CAAAGGGGTATCCATCGCCA -3’
MMP-9	P1: 5’- AGGACGGCAATGCTGATG -3’
	P2: 5’- TCGTAGTTGGCGGTCGTG -3’
E-cadherin	P1: 5’- ATTCTGATTCTGCTGCTCTTG -3’
	P2: 5’- AGTCCTGGTCCTCTTCTCC -3’
Vimentin	P1: 5’- AATGACCGCTTCGCCAAC -3’
	P2: 5’- CCGCATCTCCTCCTCGTAG -3’
N-cadherin	P1: 5’- TGTTTGACTATGAAGGCAGTGG -3’
	P2: 5’- TCAGTCATCACCTCCACCAT -3’
